# Reduced Newcastle disease virus-induced oncolysis in a subpopulation of cisplatin-resistant MCF7 cells is associated with survivin stabilization

**DOI:** 10.1186/1475-2867-12-35

**Published:** 2012-08-01

**Authors:** Mohd-Hafifi Jamal, Wei-Choong Ch’ng, Khatijah Yusoff, Norazizah Shafee

**Affiliations:** 1 Department of Microbiology, Faculty of Biotechnology and Biomolecular Sciences, Universiti Putra Malaysia, UPM Serdang 43400, Malaysia; 2 Institute of Biosciences, Universiti Putra Malaysia, UPM Serdang 43400, Malaysia

**Keywords:** Newcastle disease virus (NDV), Cisplatin resistant cells, Breast cancer, Survivin

## Abstract

**Background:**

Cisplatin resistance is a serious problem in cancer treatment. To overcome it, alternative approaches including virotherapy are being pursued. One of the candidates for anticancer virotherapy is the Newcastle disease virus (NDV). Even though NDV's oncolytic properties in various cancer cells have been widely reported, information regarding its effects on cisplatin resistant cancer cells is still limited. Therefore, we tested the oncolytic efficacy of a strain of NDV, designated as AF2240, in a cisplatin-resistant breast cancer cell line.

**Methods:**

Cisplatin-resistant cell line (MCF7-CR) was developed from the MCF7 human breast adenocarcinoma cell line by performing a seven-cyclic exposure to cisplatin. Following NDV infection, fluorescence-activated cell sorting (FACS) analysis and immunoblotting were used to measure cell viability and viral protein expression, respectively. Production of virus progeny was then assessed by using the plaque assay technique.

**Results:**

Infection of a mass population of the MCF7-CR with NDV resulted in 50% killing in the first 12 hours post-infection (hpi), comparable to the parental MCF7. From 12 hpi onwards, the remaining MCF7-CR became less susceptible to NDV killing. This reduced susceptibility led to increased viral protein synthesis and virus progeny production. The reduction was also associated with a prolonged cell survival via stabilization of the survivin protein.

**Conclusions:**

Our findings showed for the first time, the involvement of survivin in the reduction of NDV-induced oncolysis in a subpopulation of cisplatin-resistant cells. This information will be important towards improving the efficacy of NDV as an anticancer agent in drug resistant cancers.

## Background

Cisplatin *cis*-diaminedichloroplatinum(II), CDDP] is one of the widely used drugs to treat cancer patients. It was first discovered in 1969 [1] and has since been used to treat a variety of malignant tumors such as lung, ovarian, head and neck, bladder and testicular cancers [2]. Even though CDDP and other platinum complexes are not commonly used in the current therapy of breast cancers, they have recently been introduced into the clinical setting as an emerging new treatment modality [3,4]. The mode of action of this drug is believed to result from binding of its platinum molecule onto DNA of target cell. The incorporation of CDDP into DNA produces intrastrand and interstrand crosslink adducts [5,6]. The DNA-platinum adducts then prevent cells from undergoing DNA replication, prevent efficient RNA transcription and disrupting cell cycle which eventually lead them to apoptosis [7,8].

Survivin is usually found at low level in normal cells but it is highly expressed in most tumors [9]. High survivin expressions in cancer cells correlated to poor prognosis, decreased apoptosis, and increased resistance to CDDP [10,11]. As a bifunctional protein, survivin, like other members of inhibitor of apoptosis proteins (IAPs), suppresses apoptosis by binding to caspase-3, 7 and 9 [12]. Besides that, previous reports suggested that survivin played major roles in cell division where it was dominantly induced during G2/M phase to assist mitosis and cytokinesis [13].

Recently, several alternative treatment modalities have been explored to overcome the drug resistance problems, and virotherapy has emerged as a promising approach. The idea of employing virus in cancer treatment arose in the early 1960s. Since then, several groups of DNA and RNA viruses with oncolytic properties have been identified including Newcastle Disease Virus (NDV). NDV was first discovered in Newcastle upon Tyne in 1927 and it causes a highly contagious disease affecting birds and poultry [14]. The use of NDV in treating cancer was first reported by Cassel and Garret in 1965 [15] and since then, the interest in using NDV as anti-neoplastic treatment has expanded steadily. NDV strain AF2240 was first isolated in the 1960s from a field outbreak in Malaysia and it was reported to cause high mortality and morbility in poultry. A study done by Othman et al. [16] showed that NDV strain AF2240 has the ability to infect MCF7 cell line resulting in apoptosis. However, the activity of NDV AF2240 on cisplatin-resistant MCF7 cell line has not been reported. Thus, in this study, we investigated the oncolytic effect of this NDV strain on cisplatin-resistant MCF7 cancer cells.

## Results

### Cisplatin (CDDP) resistance correlates with morphological changes in MCF7 cells

**Figure 1:**
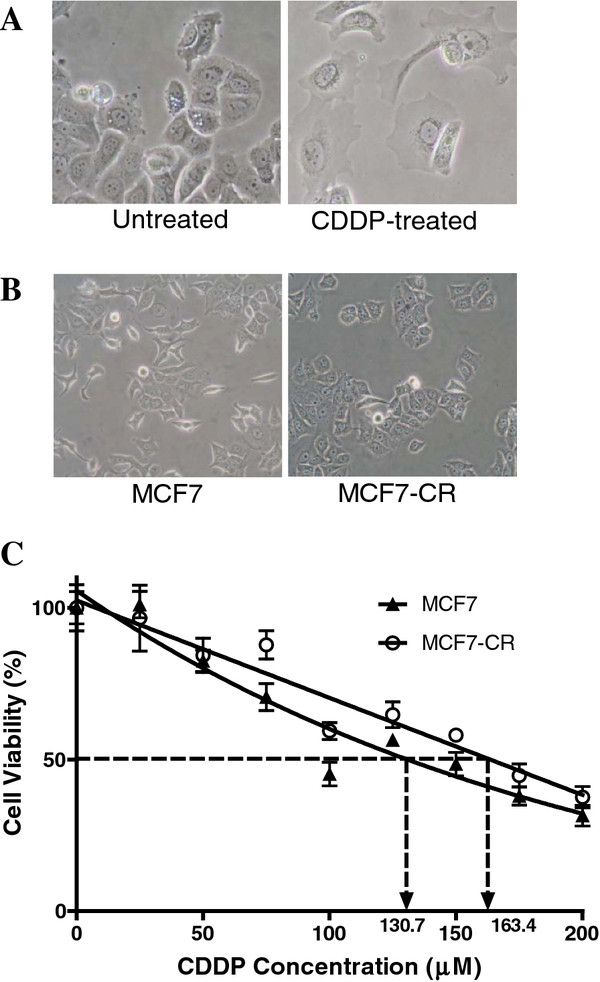
**Morphology and viability of MCF7 and MCF7-CR cells in the presence of cisplatin:** (**A**) Surviving MCF7 cells following cisplatin treatment showed an enlarged morphology (right panel), compared to the untreated cells (left panel). They were also found to attach stronger to flask surfaces (right panel). Magnification: 200×. (**B**) MCF7 cells that survived the seven-cyclic exposure to cisplatin were maintained for another 30 days in drug-free media prior to the infection experiment. The morphology of these MCF7-CR cells reverted back to the morphology of the parental MCF7. Magnification: 100×. (**C**) An increased IC_50_ was observed in the MCF7-CR compared to the MCF7 cells.

MCF7 cell line was employed as a model to study NDV oncolytic activity in CDDP-sensitive versus CDDP-resistant breast cancer cells. During the CDDP treatment, surviving MCF7 cells showed an average of 2-fold increase in size compared to the control untreated MCF7 (Figure 1A). There was no change in the DNA content of the enlarged cells (data not shown). During trypsinization, it took a longer time for them to be dislodged suggesting a stronger attachment to flask surfaces. During the 30-day maintenance in drug-free media, the surviving cells' morphology reverted back to almost similar to the parental MCF7 (Figure 1B, right panel). Prior to infection experiments, their level of resistance to CDDP was determined using the MTT assay. The resulting IC_50_ for both MCF7 and MCF7-CR was found to be 130.7 μM and 163.4 μM, respectively (Figure 1B). The newly established MCF7-CR showed 25% increase in resistance towards CDDP compared to the parental MCF-7. The resistant factor of MCF7-CR was calculated as 1.3.

### Reduced NDV-induced cytotoxicity in a subpopulation of MCF7-CR cells

**Figure 2:**
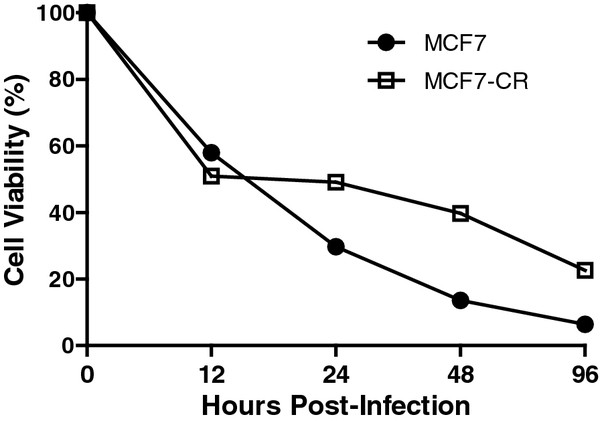
**Cell viability in the mock- and NDV-infected MCF7 and MCF7-CR cells.** Cytometric analysis of the cells was performed at 12, 24, 48 and 96 hours post-infection and viable cell populations were quantitated. The values were represented in a line graph for ease of analysis. Results are representatives of a triplicate experiments.

To investigate the effects of NDV-AF2240 cytotoxicity on MCF7 and MCF7-CR, the cells were infected with the virus at 1.0 MOI and subjected to a flowcytometric analysis at 4 different (12, 24, 48 and 96) hpi. Mock-infected cells were used as controls. For both cell lines, percent viability appeared to decrease over time (Figure 2). Even though the MCF7-CR is more resistant to CDDP than its parental MCF-7, these cells are still susceptible to NDV oncolysis. A particularly drastic decrease in cell viability was observed during the first 12 hpi, followed by a gradual decrease for the rest of the infection period. At 24 hpi, more viable cells were observed in the infected MCF7-CR compared to the MCF7. Higher viability of MCF7-CR than MCF7 was maintained until the end of the experiment. From 12 hpi to 24 hpi, percent viability in infected MCF7-CR did not change suggesting a degree of cellular resistancy towards NDV cytotoxicity in the surviving cell population.

### Increased NDV protein expression in MCF7-CR

**Figure 3:**
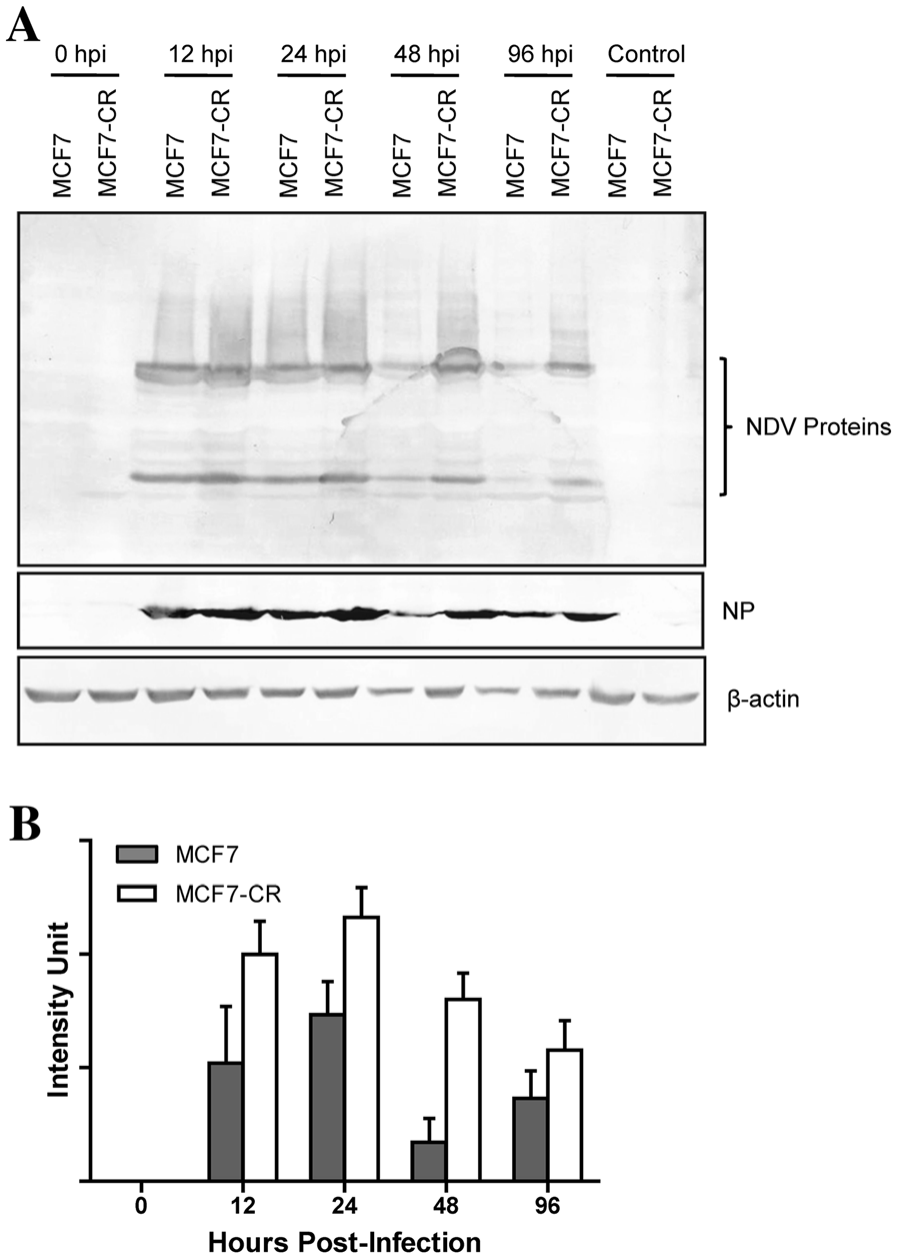
**Detection of NDV viral proteins in the mock- and NDV-infected MCF7 and MCF7-CR cells.** (**A**) Polyclonal antibodies against NDV were used to probe for viral proteins in the total cell lysates harvested following different hours post-infection. A monoclonal antibody against the NP protein of NDV was also used. (**B**) The intensity of the NP protein bands were quantitated and plotted in a graphical format. Values are presented as means ± SD of triplicate blots.

To examine whether the different pattern of cell death observed in the infected MCF7 versus MCF7-CR cells also affected NDV viral protein synthesis, total cell lysates were then subjected to SDS-PAGE and immunoblotting analyses. Mock-infected cells were used as controls. At 0 hpi, no viral protein was detected on the membrane for both the MCF7 and MCF7-CR samples (Figure 3A). After 12 hpi, several bands representing different viral proteins were observed on the membrane. This observation suggested that NDV protein synthesis occurred at high efficiency within the first 12 hours of infection. As the infection time progressed, the intensity of virus protein bands started to differ between the MCF7 and MCF7-CR.

As our objective was to study how well NDV can infect cisplatin-resistant breast cancer cells, we used cisplatin sensitive MCF7 as the benchmark. Based on the protein band intensity at 12 hpi, the MCF7-CR appeared to contain more viral proteins than MCF7 (Figure 3A). To confirm this observation, we probed the samples with a monoclonal antibody against the nucleocapsid protein (NP) of NDV. The NP protein is the most abundant protein in the virus which makes it easier to detect and analyse [17]. To investigate quantitative difference between the band intensities, the NP blot was analyzed using the ImageJ software and the intensity values were normalized against the β-actin loading control. The plotted values showed that the amount of the NP protein inside MCF7 is lower compared to MCF7-CR and their intensity differences started to increase as time progressed (Figure 3B). These results suggest that in the infected MCF7-CR, NDV protein synthesis occurred at higher level compared to the parental MCF7 cells.

### High level NDV progeny production in infected MCF7-CR

**Figure 4:**
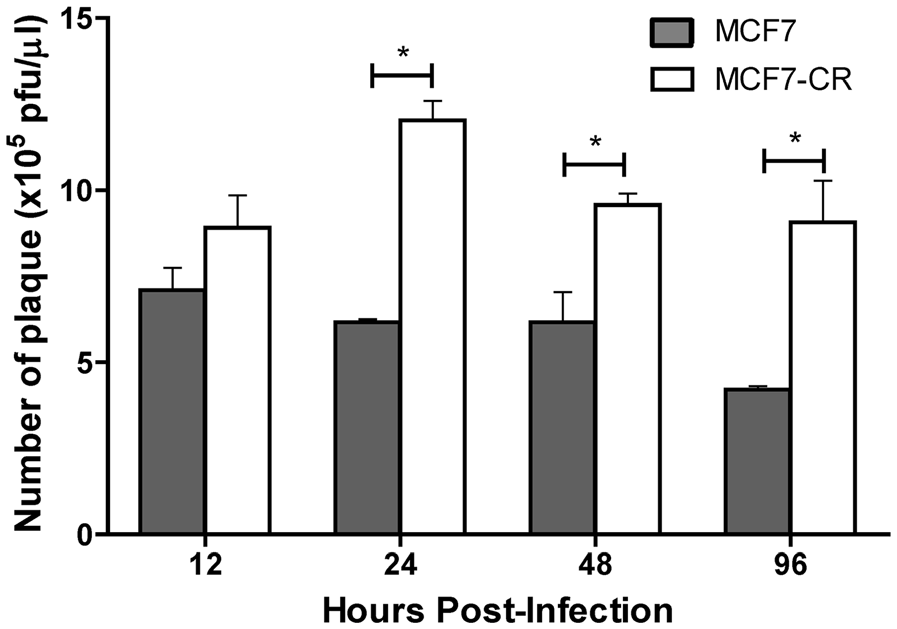
**Plaque assay analysis using the NDV-infected MCF7 and MCF7-CR cell culture media.** At 12, 24, 48 and 96 hours post-infection, 100 μl of the infected cell culture media were removed and subjected to the plaque assay as described in Methods section. The resulting plaques were calculated and a graph was plotted. Values are presented as means ± SD of triplicate experiments. *p < 0.05.

We have shown that NDV protein level was higher in the infected MCF7-CR compared to MCF7. To test whether this increase in viral protein correlates with an increase in viral progeny production, we performed a plaque assay using culture media obtained from the infected cells at 12, 24, 48 and 96 hpi. At all the time points tested, virus yield in the MCF7-CR were always higher than MCF7 (Figure 4). However, the difference was statistically significant only at 24, 48 and 96 hpi. This increase was in line with the amount of viral protein detected in the cells (Figure 3). It is interesting to note that at 24 hpi, virus yield in MCF7-CR was markedly increased compared to the other samples. This sudden jump in virus production correlated with reduced NDV-induced oncolysis (Figure 2B) and increased viral protein level (Figure 3B).

### Enhanced NDV production in MCF7-CR is associated with survivin stabilization

**Figure 5:**
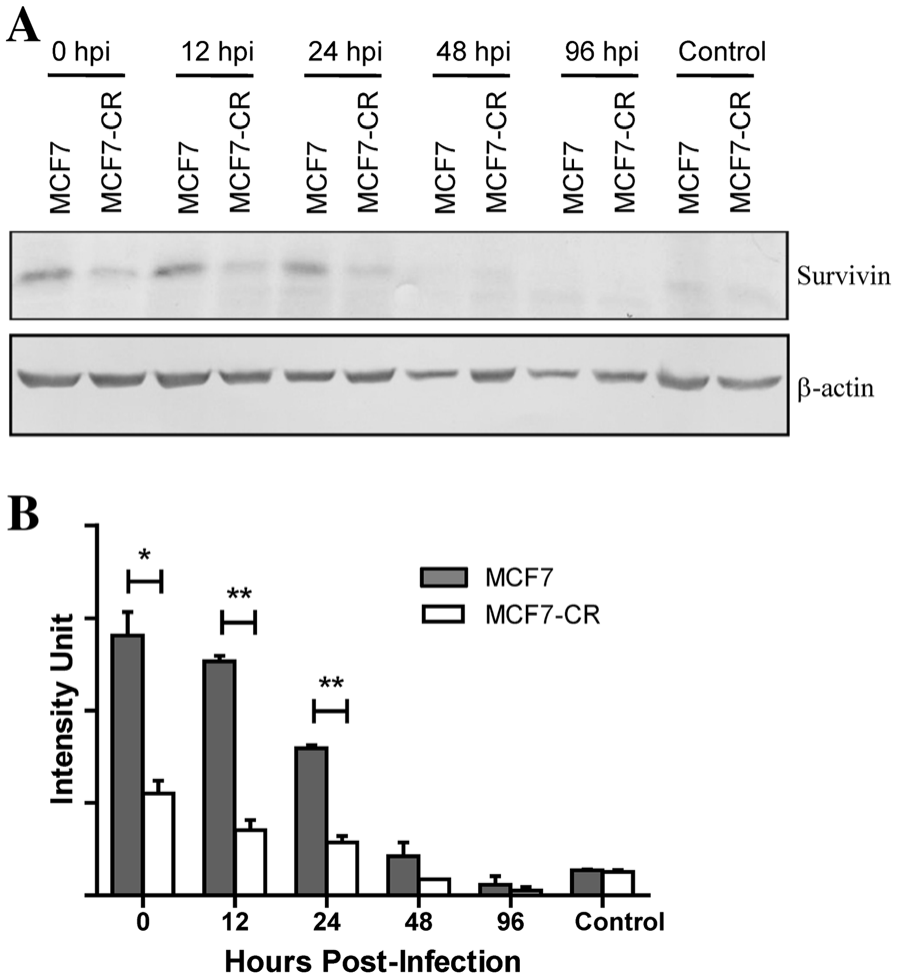
**Survivin protein levels in the mock- and NDV-infected MCF7 and MCF7-CR cells at different hpi.** (**A**) Survivin protein expression in total cell lysates were detected using a monoclonal antibody against the protein. (**B**) Intensities of the resulting survivin band were quantitated, normalized to the β-actin control and the values were plotted as means ± SD of readings from triplicate blots. *p < 0.05, **p < 0.01.

Survivin, a member of the IAP family, is highly expressed in most cancers. It has been shown to be upregulated during virus infection to assist virus production [18,19]. To test whether survivin level is affected by NDV infection particularly in MCF7-CR, we performed immunoblot studies on all the infected and mock-infected MCF7 and MCF7-CR samples. Since survivin upregulation is known to be a factor in cisplatin resistance [10,20,21], we used CDDP as a positive control in this study. Exposure of MCF7-CR but not MCF7 led to increased survivin level in the cells (data not shown). These results confirmed that our MCF7-CR responded the same way to CDDP as other previously reported cisplatin-resistant MCF7 cell lines (22). When MCF7-CR and MCF7 cells were infected with NDV, their survivin levels increased beginning immediately after the adsorption period (considered as 0 hpi in this study; Figure 5A). In certain viral infections, survivin expression was increased and it is shown to be involved in facilitating virus production [22]. At later time points after the infection, the level of survivin began to decrease. When the survivin bands were quantitated and plotted, a dramatic decrease was noted at 24 hpi in MCF7 compared to its level at 12 hpi (Figure 5B). In MCF7-CR on the other hand, the survivin level, despite being lower than MCF7, was retained. Hence, there is no significant change in MCF7-CR survivin level from 12 to 24 hpi. This retention of survivin level in MCF7-CR may contribute towards the reduced cell death (Figure 2) and increased virus production (Figure 4) at 24 hpi.

## Discussion

Cisplatin is one of the most potent and widely employed drugs in cancer treatment. However, development of resistance is a major issue in its effectiveness. Despite considerable advances in this field, the mechanism of resistance development and maintenance is still not fully understood. Alternative therapies including the use of oncolytic viruses are being studied. Recently, it was shown that a local isolate of NDV, AF2240, was able to induce high level of apoptosis in several cancer cell lines [23,24]. To investigate whether this virus is also oncolytic in cisplatin-resistant cancer cells, we studied its infectivity in MCF7 cell line versus cisplatin-resistant MCF7-CR.

To investigate the oncolytic effects of NDV-AF2240 on cisplatin-resistant cancer cells, we generated our own sub-cell line from breast cancer cell, MCF7. Repeated exposure of cisplatin to MCF7 resulted in a generation of a 1.3-fold increased in CDDP-resistance sub-cell line, MCF7-CR. This cell line displayed similar morphological changes to other cisplatin-resistant MCF7 cells [25]. Similar to these findings, our MCF7-CR also showed higher nucleus to cytoplasm ratio, and they attached stronger to flask surfaces. Following NDV infection, these cells were found to be susceptible to NDV oncolytic activity. This finding is in agreement with recent reports which showed an induction of apoptosis in chemoresistant cancer cells [26,27] using NDV-HUJ and NDV-FMW strains, respectively.

In this study, we showed that NDV AF2240 killed almost 50% of both MCF7 and MCF7-CR cell lines during the first 12 hpi. From this time point onwards, the infected MCF7 cells continued to show an exponential reduction in viability suggesting their continued susceptibility to NDV-induced killing. The MCF7-CR, however, showed no significant reduction in their viability beginning from 12 until 24 hpi. The cells which were killed during the first 12 hpi in both MCF7 and MCF7-CR perhaps represent a 'rapidly cycling' pool of cells [28]. It was shown that rapidly proliferating cells were more susceptible to vesicular stomatitis virus (VSV)-induced oncolysis [29]. In the present study, the drastic reduction in cell viability in both infected MCF7 and MCF7-CR after 12 hpi was strongly suggestive of the involvement of cell cycle phases in the infectivity and cytotoxicity of NDV. Virus internalization was previously shown to be affected by these phases [30]. In addition, cisplatin resistance caused changes in cell cycle regulatory proteins which affect their way of responding to the drug [31]. These changes perhaps allow this subpopulation of MCF7-CR to respond differently to NDV infection as well. Even though this has to be examined further, especially in the context of chemoresistant cancer cells, our results showed that certain subpopulations of CDDP-resistant cells have different degrees of susceptibility to NDV.

After 12 hpi, the surviving cells in MCF7-CR became more resistant towards NDV killing compared to MCF7. When we measured the level of viral proteins inside the cells, we found that this reduced cytotocixity in MCF7-CR after 12 hpi was associated with increased NDV protein level in the cells. Viruses are known to interfere with cell cycle regulation in order to subvert host-cell function to favor viral protein translation [26]. Even though development of cisplatin-resistance is multifactorial [7,10], these changes will undoubtedly affect the way cells respond to viral infection. One of the proposed mechanisms of cisplatin resistance development involved apoptosis inhibitor proteins (IAPs) such as survivin. Survivin expression is known to be influenced by viruses to cause cell cycle aberration in infected cells in order to increase viral protein expression [32,33]. Its expression was also increased during viral infections to facilitate virus production [22]. In this study we showed that a reduced susceptibility of MCF7-CR to NDV cytotoxicity after 12 hpi was associated with a stabilization of survivin in the cells. As the infection progressed, the survivin level became reduced which directly correlated with reduced cell viability.

The mechanism as to how NDV regulates survivin is still not completely understood. Nonetheless Zhu et al., showed that upregulation of survivin during virus infection led to a prolonged cell viability which may assist virus production [18]. In the present study, the stabilization of survivin in MCF7-CR beginning from 12 hpi might have led to their prolonged survival which allowed for increased viral protein production and viral progeny secretion. In hepatitis-B virus infection, a viral protein was shown to interact with host protein complex, affecting caspase activation in a survivin-dependent manner [19]. We observed that stabilization of survivin in MCF7-CR beginning at 12 hpi was associated with an intracellular accumulation of NDV proteins. This concomitant event may have implications in the observed cellular responses to NDV infection after 12 hpi. Based on this observation, it is tempting to speculate the involvement of viral protein(s) in the stabilization of survivin, reduction of NDV-induced cell killing and increase viral progeny secretion. We are currently investigating this possibility.

Cisplatin resistant cells have been shown to express higher survivin than their cisplatin-sensitive parents [34]. The increase in survivin rendered them less susceptible to apoptosis. These inherent properties of cisplatin-resistant cells may make them more susceptible to NDV AF2240 infectivity and viral replication. NDV-LaSota strain was shown to be more susceptible to apoptosis-resistant cells [35]. In addition, NDV-HUJ was also shown to be more infectious towards cancer cells expressing the livin protein, which is another family member of the IAPs [26]. Therefore it is likely that the increase in the intracellular viral protein and virus progeny production in MCF7-CR at 24 hpi was associated with stabilization of survivin level in the cells beginning at 12 hpi. This suggests that survivin in one of the main protein involved in the infectivity and oncolytic activity of NDV. Taken together, our findings suggest that survivin can be a potential target molecule to improve the efficacy of NDV as anti-cancer agents.

## Conclusion

Resistance to CDDP is a major problem in the effective management of cancers including metastatic breast cancers. One of the alternative approaches to address this problem is the use of oncolytic viruses. In this study, we showed that MCF7-CR cells are susceptible to NDV infection. A subpopulation of these cells became less susceptible to NDV killing after 12 hours of infection. The reduced susceptibility was associated with a stabilization of survivin which led to a prolonged cell survival leading to increased viral protein synthesis and progeny production. The reduced susceptibility of only a subpopulation of MCF7-CR cells after 12 hpi also suggests a possible relationship between NDV infection and cell cycle phases. We are currently pursuing an investigation into this relationship. Nonetheless, our findings in the current study highlight the importance of survivin in the oncolytic effects of NDV in CDDP-resistant cells. This information will be useful in the design of therapeutic approaches using NDV as an anticancer agent in chemoresistant cancers.

## Methods

### Establishment of cisplatin-resistant MCF-7 cell line

Estrogen-dependent MCF7 cells were obtained from the ATCC (HTB-22) and maintained in DMEM media supplemented with 10% fetal bovine serum and 1% antibiotic/antimycotic. The media, serum and antibiotic/antimycotic was purchased from PAA Laboratories: Pasching, Austria. A humidified atmosphere of 37°C and 5% CO_2_ was provided for cell growth. Cisplatin-resistant MCF-7 (MCF7-CR) was established from the surviving population of the parental MCF7 breast cancer cells (ATCC) following a cisplatin treatment. The treatment was performed as a seven-cyclic 24 h exposure to a final concentration of 50 mM cisplatin (Sigma-Aldrich: St. Louis, MO). Following the treatment, surviving cells were maintained for another 30 days in drug-free media. MCF-7 and MCF-7 CR cells were routinely tested for the presence of *Mycoplasma* to avoid contamination.

### Assessment of in vitro cisplatin resistancy

Cells were plated in triplicates into 96-well tissue culture plates at a concentration of 1 × 10^4^ cells per well in 200 μl of culture media. Cisplatin was added at a final concentration in the range of 0 – 200 μM 24 h post-plating and the cells were incubated for 24 h in 5% CO_2_ at 37°C. 200 μl of MTT reagent (Sigma Aldrich: St. Louis, MO; final concentration of 0.5 mg/ml in DMEM) was added to each well for 4 h incubation at 37°C, followed by addition of 100 μl of DMSO (Sigma Aldrich: St. Louis, MO). Absorbance was read at an excitation wavelength of 595 nm on an ELISA microplate reader (Model 550; BioRAD: Philadelphia, PA). Cell survival was measured as a percentage of surviving cell over untreated control cells. For both cell lines the concentration of cisplatin which resulted in 50% growth inhibition (IC_50_) was determined.

### NDV infection of sensitive and resistant MCF-7 cell cultures

Exponentially growing MCF-7 and MCF-7 CR cells were seeded in T-75 flasks at a concentration of 3 × 10^6^ cells per flask. The cells were then infected with NDV at 1 multiplicity of infection (MOI). After 1 h, virus-containing medium was removed and fresh growth medium was introduced into each flask. The cell culture was incubated at 37°C and 5% CO_2_ and harvested at different time points. Uninfected cells were used as controls.

### Harvesting of cells and Immunoblot analysis

Cell pellets were harvested by lysis in 1× RIPA buffer (Pierce Biotechnology: Rockford, IL) containing protease inhibitor cocktail (Roche Diagnostics: Mannheim, Germany) and quantitated using the BCA Protein Assay kit (Pierce Biotechnology: Rockford, IL). Equal amount of protein samples were added to 6× sodium dodecyl sulfate loading buffer (1 μl loading buffer: 5 μl protein sample). The sample mixtures were boiled for 5 min before being resolved by sodium dodecyl sulfate–polyacrylamide gel electrophoresis (SDS-PAGE) to separate the proteins on the basis of their molecular weights. After electrophoresis, the proteins were transferred to a polyvinylidene fluoride (PVDF) transfer membrane by electroblotting, and probed for survivin and NDV proteins using an anti-survivin rabbit mAb (Cell Signaling Technology: Danvers, MA), anti-actin (Sigma Aldrich: St. Louis, MO), anti-NDV and anti-NP. The blot was developed and individual bands were quantitated and normalized to the β-actin control using the ImageJ software (Wayne Rasband, NIH: Bethesda, MD).

### Plaque assay

A monolayer of SW620 cells was prepared in wells of a 6-well plate. 10-fold dilutions of virus stock were prepared and 1 ml aliquots were inoculated onto the cell monolayers. The plate was incubated at 37°C and 5% CO_2_ for 1 h. After the incubation period, the monolayers were covered with a nutrient medium containing agar and the plate was incubated for 6 days. The monolayers were fixed with 1% formaldehyde in 0.15 N NaCl and stained with neutral red for overnight. The plate was washed and dried for plaque observation.

### Statistical analysis

Statistical analyses were performed using the GraphPad Prism 5 (GraphPad Software, Inc., La Jolla, CA). Student's *t*-test was used to evaluate the significance of differences between experimental groups. Statistically-significant differences were defined as *p* values less than 0.05.

## Abbreviations

NDV: Newcastle disease virus; CDDP: *cis*-diaminedichloroplatinum(II) (cisplatin); DMEM: Dulbecco’s modified eagle medium; MTT: 3-(4,5-Dimethylthiazol-2-yl)-2,5-diphenyltetrazolium bromide; DMSO: Dimethyl sulfoxide; MOI: Multiplicity of infection; RIPA: Radioimmunoprecipitation assay; BCA: Bicinchoninic acid; SDS-PAGE: Sodium dodecyl sulfate-polyacrylamide gel electrophoresis; NP: Nucleocapsid protein; ELISA: Enzyme-linked immunosorbent assay; ATCC: American type culture collection.

## Competing interests

The authors declare that they have no competing interests.

## Authors’ contributions

NS designed the study. MJ, WC performed experiments and data analysis, NS and MJ drafted the manuscript. NS, KY revised the manuscript. All authors read and approved the final manuscript.
